# Atlanta Society of Medicine

**Published:** 1886-05

**Authors:** 


### ATLANTA SOCIETY OF MEDICINE.

Officially Reported for the ATLANTA MEDICAL AND SURGICAL JOURNAL.

STATED MEETING, March 16, 1886.

Dr. N. O. Harris, Vice-President, in the chair.

Dr. H. F. Scott read a paper on tetanus, in which he directed attention to the gravity of this disease, and to the paucity and inefficiency of medical resources for its treatment.

After remarking that there was absolutely no fundamental difference between idiopathic and traumatic cases—the symptoms of both being identical—and dwelling upon the etiology, symptomatology, etc., Dr. Scott spoke of the difficulty of differentiating between tetanus and several other affections, whose symptoms were very similar.

Only two points of difference between it and strychnine poisoning were mentioned—as the remainder would readily suggest themselves—the former being a progressive disease, while the latter appears suddenly in all its severity, there being in the first a constant stiffness of the muscles of the jaw, while in the second the jaw is only locked during the spasm.

As between hydrophobia and tetanus, the peculiar restlessness of mind and body in the former, the entire intermissions of spasms at intervals, and the well-known aversion to fluids, with the most excessive thirst present, would be sufficient to distinguish between the two; though it should be borne in mind that cases are reported where these two affections are present in one and the same case.

The fact that the patient's mental faculties remain unimpaired in tetanus—any delirium being seldom observed—would aid in distinguishing it from cerebro-spinal meningitis.

In treating tetanus it should be remembered that acute cases die from suffocation through spasm of the muscles of respiration—more particularly the laryngeal muscles. Now itis generally admitted that the disease, once well begun, will run its course; hence efforts should to a great extent be directed to preventing apnœa or suffocation.

For this purpose tracheotomy is highly recommended, and if undertaken sufficiently early seems worthy of adoption, for the reason that with a tube in the trachea, death by laryngeal spasm cannot occur, and hence there is often a better prospect of recovery, together with other appropriate treatment.

On the other hand, chronic cases prove fatal from exhaustion; here efforts must be directed to feeding the patient by the mouth if practicable; if not, by nutritious enemata repeated every four to six hours.

Morphia, internally and hypodermically, has been highly recommended; its injection deep within the drawn muscles is said to be especially beneficial.

Patients are greatly relieved by being brought thoroughly under the influence of chloroform, bearing in mind always the natural tendency to spasm of glottis and other muscles of respiration, and therefore being guarded in its administration.

But above all, the reader thought hydrate of chloral worthy of great confidence, given in large doses, per enæma if necessary. His experience with hyoscyamine in other affections led him to believe it would prove equally effective in controlling paroxysms of tetanus. It should be given hypodermically, in doses of one-sixteenth to one-tenth grain, repeated *pro re nata.*

Dr. S. remarked that he was a firm believer in the "nervous theory" of tetanus—according to which some known or unknown peripheral irritation exerts its influence upon the nerve filaments, and is thence conducted along the nerve-trunks to the spinal cord, thereby inducing a state of exaggerated functional activity in the latter, as evidenced by muscular contractions, etc.

He further desired to direct attention to the fact that in many traumatic cases the nerves supplying the injured parts, as well as their coverings, are inflamed; in other cases there is found simply hyperæmia of the neurilemma, within which blood is often extravasated. This hyperæmia and swelling, often with neuritis and perineuritis, gradually ascends the nerve, becoming more and more evident the longer the initial irritation lasts, until it reaches the cord itself, which may show similar changes in its substance and coverings, most marked where nerves leading to injured parts join the cord. Therefore assuming the nervous theory to be the correct one, he would be inclined to treat all injuries, especially in the parts like the hands and feet, that are abundantly supplied with nerves, by first carefully searching for and removing all foreign bodies, dividing at the same time any torn or injured nerves and using strictly antiseptic measures.

As soon as the least symptom of impending tetanus was manifest, either nerve-elongation, with or without crushing, or neurotomy of a portion or all of the nerves leading to the parts, should be done, with the view of thoroughly cutting off all communication between the cord and the peripheral irritation, and thus placing the parts in a state of complete functional rest by preventing continued excitation. As the object aimed at is only the temporary interruption of nervous conductibility, nerve-stretching or crushing, or both combined, would probably be most suitable, as they, if well done, would probably compass the desired end and would leave less bad after-results. Neurotomy, or division of one or all of the nerves of the affected limb, while more certain, would leave a more or less useless and paralyzed limb. Where special signs point to the implication of only one nerve, it alone should be divided. The tissues supplied by it are said to be eventually provided for through anastomosing branches.

Even where total neurectomy, or "true nervous amputation" of the limb, as it has been called, is done, if the divided ends are carefully handled and kept as near in apposition as possible, they before long (about six months) become capable of nervous communication—not by "immediate" union, for this is impossible, but by connective tissue through which the ends of the nerves, after undergoing a process of "sprouting," are eventually joined. Should they fail to do so, union might be established by fastening the ends and bringing them together by sutures.

In the event all efforts of nature, aided by the skill of the surgeon, prove unavailing, a paralyzed limb is infinitely preferable to amputation, as recommended by Larrey and Petit, which the latter declares to be a sure cure if undertaken sufficiently early.

In 1877, in a paper on this subject, after recommending neurotomy, Dr. Scott directed special attention to the urgent necessity of an early resort to this procedure before the secondary changes of the nerve-centers became too extensive and deep-seated to afterwards yield to general treatment. He further insisted upon the propriety of dividing the nerves, not as usually done, in the immediate vicinity of the injury, but as high up as possible, in order that we might get beyond the points to which the nerve changes had reached, fearing that their nerve changes, once well developed, might alone be sufficient, after all peripheral irritation was shut off to keep alive the flames of an already excited cord.

Dr. Elkin thought that the theories usually advanced as regards the cause of tetanus were not sufficient to explain all the phenomena. Most cases were of traumatic character, and if the nervous theory was correct, if the seat of the trouble was in the spinal cord, he could not see how section of the peripheral nerves could do good. He thought that of all theories the most plausible one was the theory of a specific microbe, a point in favor of which was the fact that tetanus was much more infrequent since the introduction of the antiseptic methods; that this theory could explain all the phenomena, the microbe gaining entrance into the system through fracture of the skin or mucous membrane, and when present in abundance produced its toxic effects. He disagreed with Dr. Scott as to the temperature changes, and was under the impression that there was always a rise, even as high as 107° F.; that in mild cases there was a slight rise and in grave cases high temperature. As to the sudden rise of temperature after death, he was inclined to agree with those who attributed it to an evolution of heat as a result of the coagulation of myosin.

Dr. Wile stated that, in his opinion, there was as yet little positive knowledge on this subject. He had nothing to offer as to the clinical history of tetanus, but having had experience at the *post-mortem* table in a few cases, he felt a special interest in the pathological anatomy of the subject. In these cases there was hyperæmia of the meninges of the spinal cord with some effusion. A microscopic examination of the muscles, affected with tonic spasm, showed a rupture of some of the muscular fibres. But these lesions are by no means constant, as other and more profound changes have been found by different pathologists. Rokitansky claimed that tetanus was caused by an exudation that exerted pressure on the nerve fibrils. Clarke found a granular degeneration of gray matter around the central spinal canal; whereas Michand found a central myelitis in four cases. Dr. Scott seems to favor the idea of an ascending neuritis. But there is no neuritis in idiopathic tetanus, and, like the lesions just cited, it is not a constant feature of traumatic tetanus. The speaker was of the opinion that the pathological lesions found thus far were secondary and in no way to be regarded as causative. As to the rise of temperature after death, he did not believe, with Dr. Elkin, that it was caused by coagulation of myosin, for in that case it would be of common occurrence, as *rigor mortis* is due to the coagulation of myosin. The question of the sudden evolution of heat after death was, he thought, still problematic.

Dr. Cooper did not agree with the reader of the paper in regarding an ascending neuritis as the cause of tetanus, because there are other conditions giving rise to ascending neuritis without causing tetanus, and that, furthermore, in idiopathic tetanus there is no neuritis. He believed that Bilbroth's theory of blood-poison best explained the facts, and that section of the nerve was the best thing to do to remove a source of irritation. He mentioned the symptom that Gross claimed as constant, namely, that of pain in the epigastric region, with spasm of the diaphragm, which begins early and ends with the disease.

Dr. Westmoreland, jr., failed to see how the blood-poison theory explained idiopathic tetanus, or how the microbe theory could account for the rapidity with which the disease often occurs, as Otis, in his history of the rebellion, reports that the largest number of cases occurred on the first day after amputation. He thought that the phenomena of tetanus were the result of reflex action due to peripheral irritation. He reported four cases of amputation in which tetanus appeared on the tenth day and resulted fatally. Climatic and race influences were referred to; that it occurred more among the blacks, and was more common though less fatal in warmer climates.

Dr. Gaston thought that the pathological anatomy of the disease was not fixed, and that death often occurred before there was time for much tissue change. In his experience of tetanus, the ordinary symptoms of inflammation were not present, and there was no suppuration. He believed that there was some peculiar change in the peripheral nerve fibrils, which change was propagated along the nerves to the spinal cord, and that the changes in the spinal cord were secondary. He had had experience in about twenty cases, mostly in tropical countries; and as to treatment, used chloroform to control spasm, and large doses of chloral hydrate and bromide of potash. After the disease had once developed, it was no longer local, but general, and that section of nerves was unphilosophical. He also used the warm bath, and in one case lobelia inflata, pushed to its physiological limit, had a happy effect. At intervals between the administration of the chloral and bromide, he was accustomed to give twenty-grain doses of quinine.

